# Formation of Se (0) Nanoparticles by *Duganella sp. andAgrobacterium sp.* isolated from Se-laden soil of North-East Punjab, India

**DOI:** 10.1186/1475-2859-11-64

**Published:** 2012-07-09

**Authors:** Mini Bajaj, Susan Schmidt, Josef Winter

**Affiliations:** 1 Institute of Biology for Engineers and Biotechnology of Wastewater, Karlsruhe Institute of Technology, 76131, Karlsruhe, Germany

**Keywords:** Bacteria isolation, 16 S rDNA alignment, *Duganella sp.*, *Agrobacterium sp*., Selenite reduction, SEM-EDX analysis, Biosynthesis, Se(0) nanoparticles

## Abstract

**Background:**

Selenium (Se) is an essential trace element, but is toxic at high concentrations. Depending upon the geological background, the land use or on anthropogenic pollution, different amounts of Se may be present in soil. Its toxicity is related to the oxyanions selenate and selenite as they are water soluble and bioavailable. Microorganisms play an important role in Se transformations in soil and its cycling in the environment by transforming water-soluble oxyanions into water insoluble, non-toxic elemental Se (0). For this study, soil samples were collected from selenium-contaminated agricultural soils of Punjab/India to enrich and isolate microbes that interacted with the Se cycle.

**Results:**

A mixed microbial culture enriched from the arable soil of Punjab could reduce 230 mg/l of water soluble selenite to spherical Se (0) nanoparticles during aerobic growth as confirmed by SEM-EDX. Four pure cultures (C 1, C 4, C 6, C 7) of Gram negative, oxidase and catalase positive, aerobic bacteria were isolated from this mixed microbial consortium and identified by 16 S rDNA gene sequence alignment as two strains of *Duganella sp.* (C 1, C 4) and two strains of *Agrobacterium sp.*(C 6, C 7). SEM/TEM-EDX analyses of the culture broth of the four strains revealed excretion of uniformly round sharply contoured Se (0) nanoparticles by all cultures. Their size ranged from 140–200 nm in cultures of strains C 1 and C 4, and from 185–190 nm in cultures of strains C 6 and C 7. Both *Duganella sp.* revealed better selenite reduction efficiencies than the two *Agrobacterium sp.*

**Conclusions:**

This is the first study reporting the capability of newly isolated, aerobically growing *Duganella sp.* and *Agrobacterium sp.* from soils of Punjab/India to form spherical, regularly formed Se (0) nanoparticles from water soluble selenite. Among others, the four strains may significantly contribute to the biogeochemical cycling of Se in soil. Bioconversion of toxic selenite to non-toxic Se (0) nanoparticles under aerobic conditions in general may be useful for detoxification of agricultural soil, since elemental Se may not be taken up by the roots of plants and thus allow non-dangerous fodder and food production on Se-containing soil.

## Background

Selenium (Se) is an essential trace element and a constituent of selenoproteins which act as antioxidants, playing an important role in the protection of cellular damages from oxygen radicals and preventing the development of chronic ailments like cancer and heart diseases [1]. Inspite of health benefits of Se at low concentrations, Se is highly toxic if its recommended daily dietary intake by adults exceeds the limit of 400 μg/d, causing selenosis [2]. Carbon shales, phosphotic rocks and coal are rich natural sources of Se in the environment [3]. Se concentrations ranging from 0.01 to 1200 mg/kg can be found in soils due to a number of factors such as the Se content of the parent rock, deposition of mining residues, magma and ashes from volcanic eruptions or seleniferous erosion materials or of residues from fossil fuel combustion. Also poor drainage, irrigation with Se-containing water and fertilization with Se-containing phosphate as well as topographic and climate conditions may be responsible for the elevated Se concentrations in soils [4]. Besides the inorganic compounds of Se in mineral fertilizers, some organic selenium compounds also find their use in agriculture due to their bactericidal, fungicidal and herbicidal properties [5]. Within the four inorganic Se oxidation states (−II, 0, +IV, +VI) found in nature, the oxyanions selenate and selenite are most mobile and detrimental as they are bioavailable and can easily be taken up by the plants from Se-rich soil or Se-containing irrigation water and thus enter the plant-animal/human food chain, posing a health risk for animals and humans [4,6]. Diseases, such as skin lesions and hair fall as a result of consuming water or plants grown in north-east Punjab, India have been associated with the high Se concentration in soil and irrigation water of that area [4,7]. The sources of Se in soil of that region are still unknown, but the high Se concentrations might, at least in part, stem from the use of Se-containing groundwater for irrigation and the practice of rice-wheat crop rotation. It was found in our previous study [4] that microbes in the top soils of Jainpur village in Punjab, which has the highest total Se concentration (up to 11.6 mg/kg) among all the collected sediment samples, have gained resistance to high soluble Se concentrations and could reduce selenate or selenite to Se (0). Due to the toxicity and changing bioavailability of selenium, most of the previous investigations were focused on the permanent removal or immobilization of selenium oxyanions by physical, chemical or biological processes. A permanent removal could be obtained by stimulating microbes for methylation of Se to generate volatile Se-compounds in soil or through direct or gravity filtration of insoluble elemental selenium (Se^0^) in water after microbial reduction of Se compounds [8]. Soil bacteria play an important role in Se transformations from soluble toxic forms [Se (IV), Se (VI)] to insoluble non-toxic Se (0) as part of a detoxification mechanism which could well be exploited for bioremediation. The full redox cycle of Se in nature is dependent on geochemical as well as on microbial transformations by soil bacteria [3,4]. These microbes could be exploited for periodic or permanent avoidance of Se toxicity, since the rate of Se (IV) or Se (VI) reduction is higher than that of Se (0) oxidation [9]. A number of anaerobic and anoxic bacteria e.g. *Geobacter sulfurreducens, Shewanella oneidensis, S. sp. HN-41, Veillonella atypica, Rhodospirillum rubrum, Sulfurospirillum barnesii, Bacillus selenitireducens* and *Selenihalanerobacter shriftii*[6,10-13] have been identified to form Se nanoparticles by reducing Se oxyanions to elemental Se (0) during the biogeochemical cycling of Se. To date the main research focus was laid on anaerobic Se reduction. Due to this, the variety of Se nanoparticle-forming aerobic bacteria is less known compared to anaerobic/anoxic bacteria and is confined mainly to species of *Pseudomonas* and *Bacillus* such as *P. fluorescens, P. aeruginosaB. subtilis* or *B. megaterium*[14-16]. Se nanoparticles are regarded as promising material for a number of applications in particular for the photovoltaic and semiconductor industry due to their high particle dispersion and unique electrical and optical properties [13,17]. Because of their high activity in biological tissues and low toxicity, Se nanoparticles are getting attention for medical applications. Nano-Se has exhibited novel in vitro and in vivo antioxidant activities through the activation of seleno enzymes, and as chemo-preventive and -therapeutic agents [18]. Se nanoparticles inhibit growth of *Staphylococcus aureus* and it has been suggested to use them as human medicine for preventing and treating *S. aureus* infections [19]. Besides this, Se nanoparticles could be used to remove metallic pollutants like copper from aqueous solutions [17].

As microbes apparently play an important role in the biogeochemical cycle of Se in natural environment, Se resistance of indigenous aerobic microbes in Se rich sediments of the Punjab region in India and biotransformation of selenite to non toxic elemental Se (0) were investigated in this study. From the mixed microbial culture that was enriched in the presence of Se, four strains of bacteria were isolated and identified. Both, the mixed culture as well as the two *Duganella sp.* and the two *Agrobacterium sp.* isolates were capable of producing exogenous Se (0) nanoparticles by reducing Se (IV) under aerobic conditions. Generation of Se (0) nanoparticles by strains of these two genera has not been reported previously. *Duganella* is a rarely described genus with the most well known species *D. violacienigra,* a violet-black pigmented bacterium isolated from forest soils in China [20].

In this study we report the isolation of pure cultures of two *Duganella* species and two *Agrobacterium* species from Se rich sediments of Punjab. Genus assignment of the isolates was done by 16 S rDNA alignment, Se (IV) reduction by ion chromatography and qualitative examination of biosynthesized nanoparticles with scanning and transmission electron microscopy (SEM and TEM) and by energy dispersive X-ray spectroscopy (EDX).

## Results and Discussions

### Enrichment of aerobic selenite reducing bacteria from soil of the Punjab area in India

**Figure 1:**
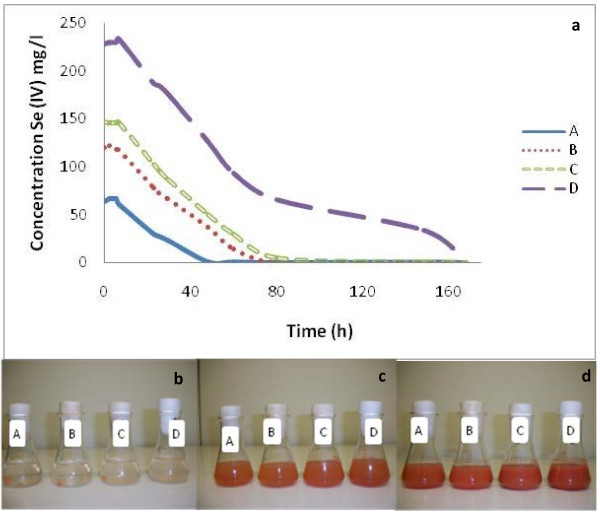
**Selenite reduction by the mixed microbial culture isolated from agricultural soil.** Selenite reduction at different Se (IV) concentrations (**a**) and development of red coloration in cultures after 5.5 h (**b**), 23 h (**c**) and 48 h (**d**) of incubation.

**Figure 2:**
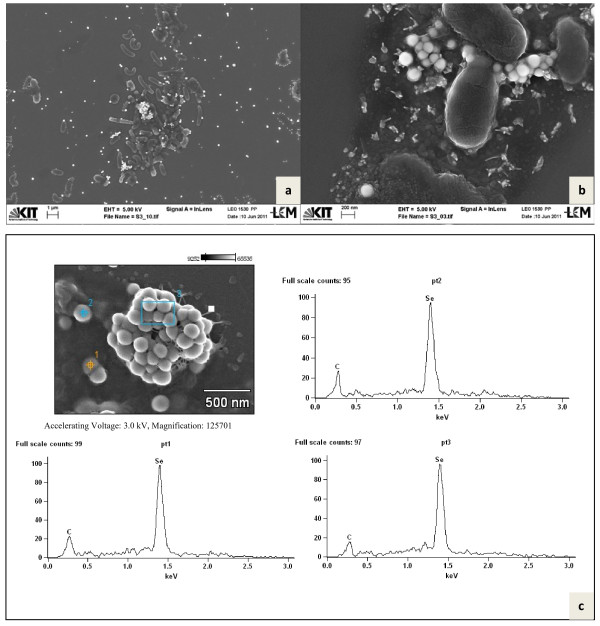
**Scanning electron microscopy of mixed cultures**. SEM of the mixed culture of bacteria (**a**) and at higher magnification along with bright round Se (0) nanoparticles (**b**). SEM-EDX spectrum of 3 targeted points of nanoparticle agglomerates, entrapped in EPS (**c**).

From a soil slurry in enrichment medium (EM) a mixed culture free of soil particles was obtained after several transfers into fresh EM that could grow in the presence of 40 mg/l of Se (IV), or reduce this amount of Se (IV) in less than 2 days. Then, 2.5% (v/v) of this cell suspension was inoculated into EM with increasing Se (IV) concentrations. The medium started to turn red within 2–3 hours of incubation, indicating the formation of red-colored elemental Se (0). Ion chromatographic analyses revealed disappearance of Se (IV) ions and no significant formation of Se (VI) ions. This was taken as an indication for the absence of selenite oxidizing bacteria in the mixed culture and a reduction of selenite to Se (0). Oxyanion reduction assays with this microbial consortium (1.25% v/v inoculum in EM) were carried out for different concentrations of selenite ranging from 65 to 230 mg/l Se (IV) ions. While the decrease of Se (IV) gave reasonable curves (Figure 1), the red color of Se (0) apparently interfered with the optical density measurement due to which no apprehensive bacterial growth data could be recorded. No change of the Se concentration was observed in sterile controls (data not shown) confirming the biotic reduction of selenite in assays with microorganisms. The time requirement for reduction of 100% Se (IV) increased with increasing initial Se (IV) concentration: It took 48 h for reduction of 64 mg/l Se (IV) and a week for reduction of 230 mg/l Se (IV) (Figure 1). The Se (IV)-reducing enrichment culture was maintained further by sequential transfers of 2.5% inoculum into fresh, 160 mg/l Se (IV)-containing EM. When culture suspensions were analyzed with SEM, spherical nanoparticles were present in the culture broth (Figure 2a). At the time of analysis after 68 h incubation, 87% of 160 mg/l Se (IV) was reduced. The nanoparticles seemed to be abundantly entrapped in slimy, extrapolymeric substances (EPS) around single cells or were agglomerated in the neighborhood of bacteria (Figure 2b). Only a few spheres seemed to float freely. SEM-EDX spectra confirmed that these particles were composed of elemental selenium (Figure 2c). The size of the spherical Se (0) nanoparticles ranged from 100–220 nm. Formation of large crystals of elemental selenium during biogenic selenite reduction was apparently prohibited due to the presence of proteins. These proteins play functional roles in Se reduction in spatial association with the formed biominerals [21]. In the same culture some irregularly formed particles with a diameter of 20–30 nm were also observed but no EDX signals of these particles were detected. They were assumed to be the crystals of salts present in the EM. The enrichment culture was also grown in the presence of 40–100 mg/l selenate (Se VI), but no reduction or change to red color was observed during growth after a prolonged period of incubation. All or at least some members of the microbial consortium were resistant to selenate and grew with glucose, as could be observed by phase contrast microscopy and by an increase of the optical density (results not shown).

### Isolation of pure cultures, phylogenetic identification and selenite reduction

**Figure 3:**
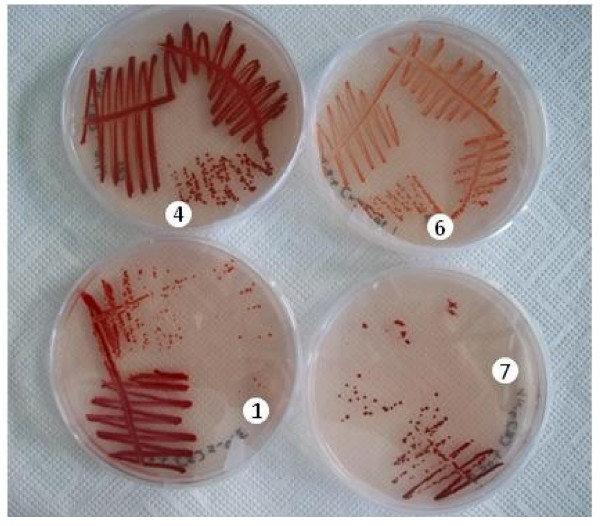
**Colonies of four strains on agar plates showing selenite reduction to red-colored elemental selenium.** The strains C 1, C 4, C 6 and C 7 are represented as 1, 4, 6 and 7, respectively, in the illustration.

Pure cultures of four strains (C 1, C 4, C 6 and C 7) were isolated from the enrichment culture by sequential transfers of liquid cultures onto 160 mg/l Se (IV)-containing EM-agar plates. Each of the four strains formed orange-red to bright red colonies on the agar plates (Figure 3). The isolated strains were identified phylogenetically by sequencing their 16 S rDNA gene and alignments of the sequences with reference strains that have been deposited in the National Center for Biotechnology Information (NCBI) database by using Basic Local Alignment Search Tool (BLAST). Strain C 1 (1089 bases) had 97% sequence similarity with *Duganella violaceinigra strain YIM 31327* and 98% with *Duganella sp. MICO-C*. The 16 S rDNA sequence of strain C 4 (1346 bases) had 95% similarity with *Duganella violaceinigra strain YIM 31327* and 98% similarity with *Duganella sp. MICO-C* and an uncultured beta proteobacterium, *clone LJ-J183,* respectively. The alignment of partial sequences of strain C 1 with those of strain C 4 showed 5.8% dissimilarities including gaps in the compared 1070 bases. Therefore it is ruled out that they are the same species. Both strains did not reveal the typical violet-black colonies of *D. violaceinigra*[20], indicating that they were different. The genus *Duganella* was first described by Hiraishi et al. [22] by reclassification of *Zoogloea ramigera IAM 12670* as *Duganella zoogloeoides.* Since then only one species, *D. violaceinigra*[20,23], was added to this genus. Most recently Kämper et al. [23] have described a new species, *Duganella phyllosphaerae*, and at the same time recommended to reclassify *D. violaceinigra* into the novel genus, *Pseudoduganella violaceinigra comb. nov.* They argued with a low 16 S rDNA gene sequence similarity of < 97% and pointed out significant differences in phenotypic and chemotaxonomic properties between *D. zoogloeoides* and the new species *D. phyllosphaerae*. The isolates C 1 and C 4 share phenotypic characteristics with *Duganella* species (Table 1), but for a detailed phylogenetic classification analysis of cellular fatty acids, ubiquinons, the predominant polar lipids and DNA-DNA hybridization are necessary. Apart of that, both strains seem to belong to the genus *Duganella,* within the class of β-Proteobacteria, family of Oxalobacteraceae, which comprises the Gram negative strict aerobic/anaerobic bacteria. None of the described *Duganella* sp. was previously investigated for their tolerance to grow in the presence of high selenium concentrations or to reduce Se (IV) to Se (0).

**Table 1:** Phenotypic characteristics of four strains isolated after enrichment from Se-containing soils in north-east Punjab, India

**Parameters** (Growth in)	**C1**	**C4**	**C6**	**C7**
Liquid medium after 7 d	Single suspended cells		Clump formation	
Nutrient agar	very good: white-yellow, flat colonies, 2–4 mm Ø	very good: white-yellow, flat colonies with dark raised center, 2–4 mm Ø	very good: white-yellow, flat colonies up to 3 mm Ø	
Tryptic-Soy agar	very weak: orange-yellow, round colonies upto 1.5 mm Ø	no growth	very good: orange-yellow, round, little slimy colonies with raised yellow center and colorless edge upto 4 mm Ø	very good: orange-yellow, round, little slimy colonies with raised yellow center and colorless edge upto 2.5 mm Ø
Yeast-Malt agar	very good: white colonies with flattened center upto 2 mm Ø	very good: white colonies with flattened center upto 3 mm Ø	very good: yellowish-white, very slimy colonies with a dark center upto 4 mm Ø	
Yeast-Mannitol agar with 0.025% Congo red			very good: slimy colonies, pink color > confirm *Agrobacterium sp*. as according to Bergey’s manual *Rhizobium sp.* forms white colonies on this medium	
1/10 Tryptic-Soy broth + 100 μg/ml Cycloheximide (OD 578 nm)	0.477	0.467	0.716	0.618
Spore forming agar	very good: no spores observed under microscope			
Growth on SP medium	−	+/−	+	+
**Enzyme activity**				
α-Galactosidase	−	−	+	+
β-Glucuronidase	−	−	+/−	+
β-Galactosidase	−	+	+	+
β-Fucosidase	−	+	+	+
β-Glucosidase	−	−	+	+
Chitinase	+/−	+	+	+
Oxidase	+	+	+	+
Catalase	+	+	+	+
Gram-staining	−	−	−	−

All four strains did not form acids from L(+)-Arabinose, D(−)-Fructose, D(+)-Galactose, D(+)-Glucosamin, D(+)-Glucose, D-Glucuron acid, Glycerin, D(+)-Glycogen, meso-Inositol, Lactulose, D(+)-Maltose, Melibiose, L(+)-Rhamnose, D(−)-Ribose, D(−)-Salicin, L(−)-Sorbose, D(+)-Xylose. Only strain C 4 formed acids from D-arabinose.

The 16 S rDNA gene sequence of strain C 6 (1352 bases) showed 97% sequence similarity to that of *Agrobacterium tumefaciens strain JDC-49* and 98% to that of *Rhizobium sp. SYF-5*, respectively, while the 16 S rDNA gene sequence of strain C 7 (1294 bases) also showed 97% sequence similarity to *Agrobacterium tumefaciens strain JDC-49.* The next highest sequence similarity with only 94% was found with *Agrobacterium tumefaciens strain IAM 1204.* The alignment of the 16 S rDNA gene sequences of strain C 6 with strain C 7 showed only 88.2% sequence similarity, confirming that C 6 and C 7 were genetically different. It is well known that *Agrobacterium* and *Rhizobium* are closely related. Phenotypic as well as 16 S rDNA sequence analyses had revealed difficulties in distinguishing these two genera as separate monophyletic clades, due to which an amalgamation of these two genera has been suggested [24]. Nevertheless, the genome structure and some phenotypic characteristics clearly set *Agrobacterium* apart from other members of the family *Rhizobiaceae*[25]. According to Bergey’s Manual of Systematic Bacteriology [26], one important test to distinguish between the genera *Agrobacterium and Rhizobium* is growth on 0.025% Congo red-containing yeast extract-mannitol agar (YEMA). When grown on this medium *Agrobacterium* forms large and stained colonies, whereas *Rhizobium* forms only small, white, translucent colonies. Both of our strains, C 6 and C 7, formed slimy pinkish red colonies on YEMA indicating that they do not belong to the genus *Rhizobium* but more likely to the genus *Agrobacterium* (Table 1). *Agrobacterium sp*. are Gram negative soil bacteria and are well known for their ability of horizontal gene transfer to plants. Hunter et al. [27] isolated a selenite reducing *Rhizobium* sp. from a laboratory bioreactor treating simulated groundwater and classified it as *R. selenireducens sp. nov.* This bacterium was related to but was genetically divergent from *R. radiobacter* (syn. *Agrobacterium tumefaciens*) or *R. rubi* (syn. *A. rubi*). No report is available describing selenite reduction by *Agrobacterium species* except for a brief note of selenate and selenite reduction by *A. tumefaciens* in a study investigating in situ Raman and X-ray spectroscopy to monitor microbial activities under high hydrostatic pressure [28]. Interestingly the two genera to which our four Gram negative isolates (C 1, C 4, C 6, C 7) from Se-rich soil in India belong were not mentioned by Ghosh et al. [29], who isolated 8 strains of Se-tolerant bacteria from Se-contaminated sediments of three different regions in India. One of these regions was the present study area. All of the newly isolated strains were catalase and oxidase positive and were tested for other phenotypic characteristic as presented in Table 1. The 16 S rDNA gene sequences of all four strains have been submitted to the GenBank at NCBI. The accession numbers are JQ745646 for strain C 1 (*Duganella sp*.), JQ745647 for strain C 2 (*Duganella sp*.), JQ745648 for strain C 6 (*Agrobacterium sp.)* and JQ745649 for strain C 7 (*Agrobacterium sp.).*

**Figure 4:**
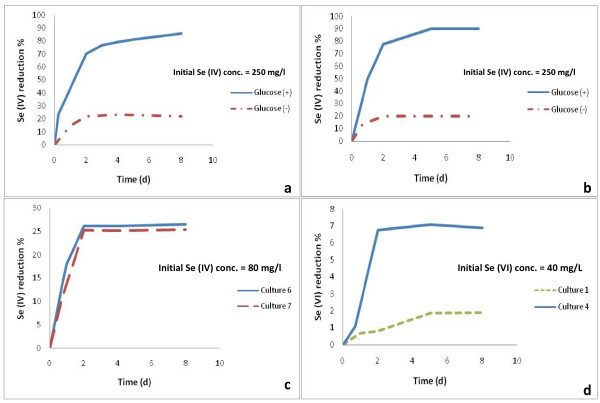
**Selenite reduction in the absence of glucose and selenate reduction by different strains**. Se (IV) reduction by strains C 1 (**a**) and C 4 (**b**) in the presence and absence of glucose and by strains C 6 and C 7 in the presence of glucose (**c**). Figure 4**d**shows reduction of Se (VI) by strains C 1 and C 4.

During growth of *Duganella sp.* with glucose in the presence of selenite, strains C 1 and C 4 reduced 81.2% and 90% of 250 mg/l Se (IV), respectively, in 5 days. In absence of glucose only 22.4% and 20% of Se (IV) were reduced initially and no further reduction occurred after prolonged incubation up to 8 days (Figure 4a and 4b). When selenate was supplied, these cultures could reduce only 2% and 7% of 40 mg/l of Se (VI) without accumulation of selenite as no Se (IV) peaks appeared in the ion chromatogram (Figure 4d). However, other intermediates or end products may have been formed during the Se-detoxification process such as volatile alkyl selenides [30]. This could be one of the reasons why no selenite was accumulating. The other possibility might be a rapid reduction of selenite to Se (0), since the bacteria were well adapted to Se (IV) reduction.

The *Agrobacterium* isolates, strains C 6 and C 7, were also grown with glucose in the presence or absence of selenite. During growth they reduced 26% and 25% of 80 mg/l Se (IV) in 2 days, respectively and no further reduction occurred after prolonged incubation (Figure 4c). When grown in the absence of glucose no Se (IV) at all was reduced by both cultures (not shown). This suggested that in the absence of glucose, storage material such as glycogen or poly hydroxyl butyrate in strains C 1 and C 4 might have delivered a restricted amount of reducing equivalents for some Se (IV) reduction and that strains C 6 and C 7 lack storage products. An exponential decrease of selenite by all strains without a lag phase was observed when glucose was present to deliver reducing equivalents (Figure 4).

### Electron microscopy and EDX analysis of pure cultures

**Figure 5:**
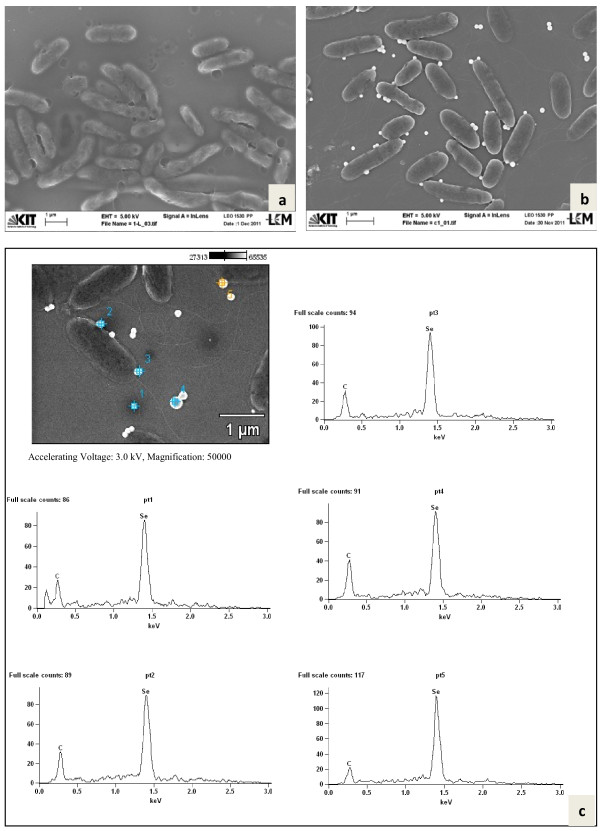
**Nanoparticles formation by strain C 1.** SEM of strain C 1 (*Duganella* sp.) grown without selenite (**a**) and in the presence of selenite, forming extracellular Se (0) nanoparticles (**b**). Figure 5c shows SEM- EDX spectra of 5 targeted points in the insert, all confirming Se (0) nanoparticles in cultures of C1.

**Figure 6:**
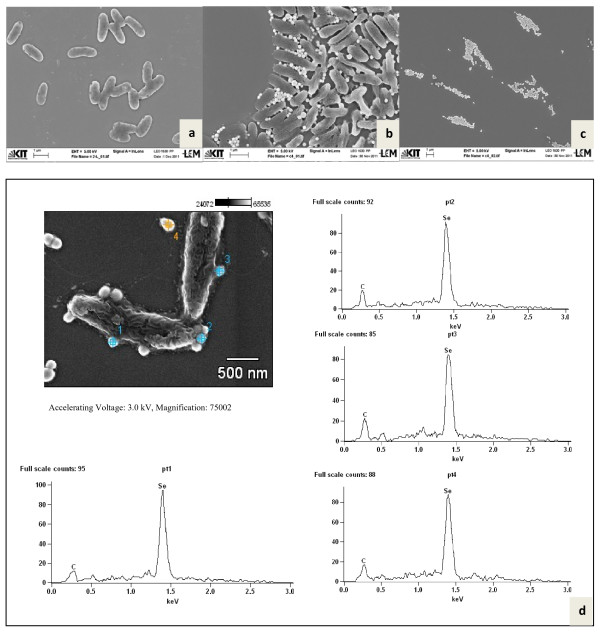
**Nanoparticle formation by strain C 4.** SEM of *Duganella* strain C 4 grown without selenite (**a**) and in the presence of selenite (**b**). Figure 6c shows agglomerates of Se nanoparticles. Figure 6d shows SEM- EDX spectra of the Se (0) nanoparticles from 4 targeted points in insert.

**Figure 7:**
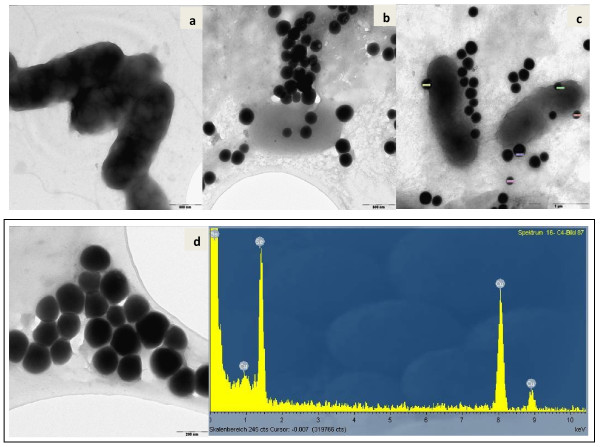
**Transmission electron microscopy of nanoparticles formed by strain C 4.** TEM of *Duganella* strain C 4 grown without selenite (**a**) and in the presence of selenite (**b**, **c**), showing Se (0) nanoparticles (some of them specified with diameter) along with bacteria and TEM-EDX spectrum of nanoparticles (**d**) confirming Se (0) nanoparticles.

**Figure 8:**
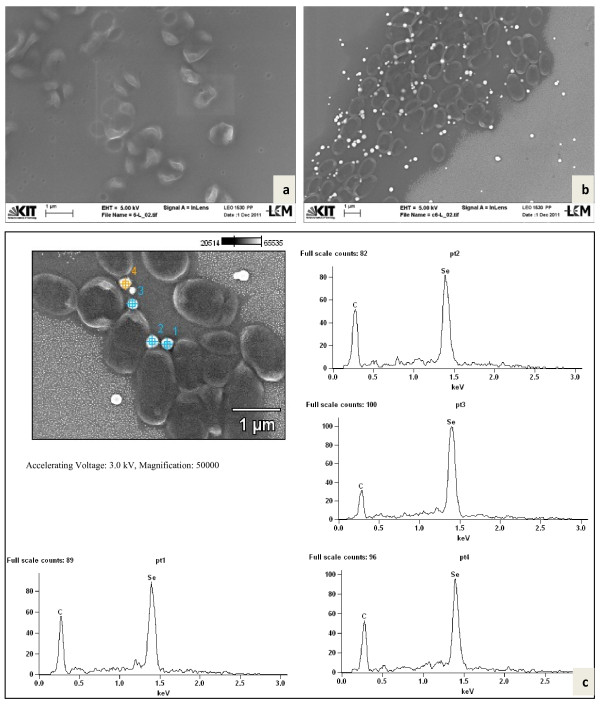
**Nanoparticle formation by strain C 6.** SEM of strain C 6 (*Agrobacterium sp.*) grown without selenite (**a**) and in the presence of selenite (**b**), showing nanoparticles along with bacteria. SEM- EDX spectra of 4 targeted points (**c**) - all confirming selenium (0) nanoparticles.

**Figure 9:**
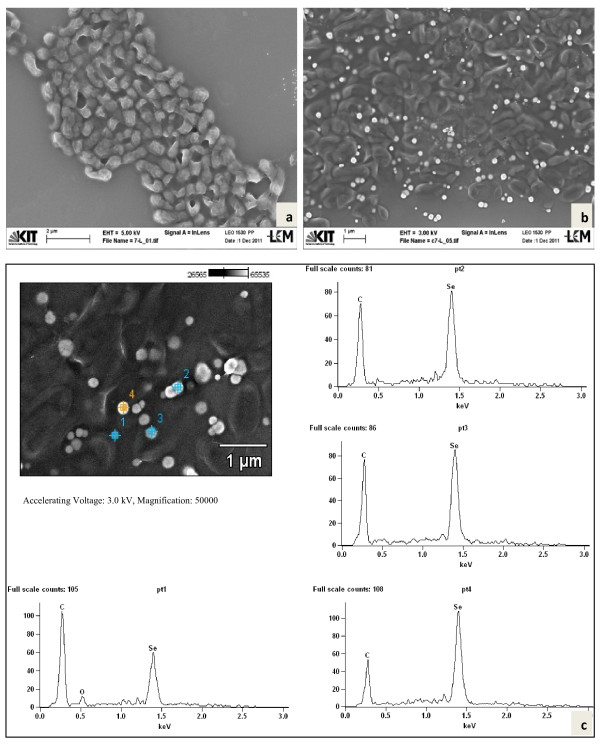
**Nanoparticle formation by strain C 7.** SEM of C 7 (*Agrobacterium sp.*) grown without selenite (**a**) and in the presence of selenite (**b**), showing nanoparticles along with bacteria. Figure 9c shows SEM- EDX spectra of 4 targeted points confirming selenium (0) nanoparticles.

Both *Duganella* strains C 1 and C 4 were rod-shaped and flagellated. They formed round spherical Se-nanoparticles, located at the cell surface and presumably embedded in extracellular polymeric substances (EPS) as well as Se nanoparticles freely floating in the medium (Figure 5, strain C 1 and Figure 6, C 4). EDX analyses of both types of nanoparticles confirmed that they consisted of Se (0) (p1 – p5 in Figure 5 and p1 – p4 in Figure 6). For additional confirmation, TEM-EDX was performed in a culture suspension of strain C 4 (Figure 7). The copper peaks in the TEM-EDX spectra were due to the Cu support grid and Be was an instrumental interference. No other peaks were observed, indicating that the nanoparticles were solid Se structures. The size of nanoparticles of strain C 1 ranged from 71 to 229 nm with the majority of the particles having a diameter of 150–200 nm. At the time of analysis, C 1 was incubated for 48 h resulting in 96.4% reduction of 160 mg/l of Se (IV). The size of Se-nanoparticles that were generated by strain C 4 ranged from 107–246 nm with most particles having a diameter of 140–180 nm. The formation of perfect round shaped Se-nanoparticles outside of cells was also observed by the *Agrobacterium* strains C 6 and C 7, which formed agglomerated spheres when they were detached from the cell surfaces. EDX analyses revealed that the nanoparticles consisted of Se (0) (Figures 8,9). The larger peaks of carbon in SEM-EDX spectra of *Agrobacterium* strains C 6 and C 7 (Figures 8,9) compared to those of strains *Duganella* C 1 and C 4 (Figures 5,6) may indicate the presence of more EPS around the nanoparticles of strains C 6 and C 7, which already tended to clump formation during growth in liquid media. In cultures of both isolates of *Agrobacterium sp. strains* C 6 and C 7 only a few freely floating nanoparticles were seen as compared to the *Duganlla sp. strains* C 1 and C 4. This may have been due to the much lower extent of selenite reduction by strains C 6 and C 7 (20% and 23% of 160 mg/l Se (IV)) at the time of SEM with the majority of selenite still being in solution. If no bacterial inoculum was added to the selenite-containing medium or if all four strains were grown in media without selenite addition, Se nanoparticles were not found (Figures 5,6,7,8,9) confirming the biogenesis of Se nanoparticles from selenite. In cultures of strain C 6 the size of Se nanoparticles ranged from 120–300 nm, most commonly ~185 nm and in cultures of strain C 7 it was from 80 – 257 nm, most commonly ~ 190 nm. The particle size in our study was smaller than the anaerobically formed nanoparticles of 200–400 nm diameter synthesized by *Sulfurospirillum barnesii, Selenihalanaerobacter shriftii* or *Bacillus selenitireducens*[11]. In the study of Oremland et al. [11] chemically reduced Se (IV) produced a vitreous, black allotrope, consisting of aggregates of various dimensions with extremely variable particle size distribution, whereas nanoparticles formed by chemical oxidation of H_2_S produced unstructured amorphous aggregates in the range of 200–800 nm. In our study, almost all the nanoparticles were perfect round spheres which seem to be a commonly observed feature of biosynthesized nanoparticles, as evident from other studies [13,14,31]. Debieux et al. [31] isolated a protein SefA which plays a functional role in stabilizing Se nanospheres and thus their shape during bacterial reduction of selenite. It is assumed that the majority, if not all Se nanoparticles were formed extracellularly, since they were found attached singly or as agglomerates to the outer cell membrane. Moreover, Se nanoparticles were too large to be released from bacterial cells without rupturing the cell walls which appeared to be intact as observed during SEM/TEM analysis. However, bacterial cells of strain C 6 and particularly of strain C 7 appeared somewhat out of shape (both with and without Se in growth medium) that might have been due to cell shrinkage by excessive moisture loss during SEM analysis, although mounts were only air dried. Furthermore, the same procedure was applied for strains C 1 and C 4 which retained their shape indicating different level of sensitivities of bacteria towards drying. The mechanism of aerobic selenite reduction is not yet fully understood. It is assumed that the electron shuttle enzymatic metal reduction is a NADPH-dependent reductase process [32]. While investigating aerobic selenite reduction by *Pseudomonas fluorescens,* Belzile et al. [33] suggested that NADPH generated by malic enzyme is used for electron transfer during aerobic Se (IV) reduction, which could take place either in the cytoplasm or periplasm of cells and the resulting elemental selenium is processed for its extrusion to the cell surface.

## Conclusions

Not many studies are available describing aerobic Se reduction and production of Se (0) nanoparticles in comparison to investigations on anaerobic/anoxic reduction. In this study, two strains of *Duganella sp*. and two strains of *Agrobacterium sp*. were isolated from Se-polluted agricultural soils of Punjab, India. The four strains were able to reduce selenite under aerobic conditions producing Se nanospheres on the cell surface as indicated by SEM/TEM-EDX analyses. The *Duganella* strains C1 and C4 could reduce selenite more rapidly than the *Agrobacterium* strains C 6 and C 7. To date no *Duganella* species were reported to have the capability to reduce selenite to Se (0) and similar studies with *Agrobacterium* have rarely been undertaken. The formation of uniformly round Se (0) nanoparticles by these bacteria under aerobic growth conditions may serve for detoxification and is of particular interest for nanoparticle production, as aerobic cultures could easily be handled. Further research is required to investigate the stability of these biosynthetic nanoparticles in different environments, their quantitative separation from biomass and EPS and their catalytic reactions. In ecosystems with a high geogenic background of Se compounds, the ability of indigenous soil bacteria to reduce soluble selenite to insoluble and thus non-toxic Se(0) could be a means to prevent Se uptake by plants for fodder and food production. If selenite-reducing bacteria in the rhizosphere could be activated at least during the cropping season to inactivate the selenite in the soil and from irrigation water at least a periodical “bioremediation” could be maintained in selenite containing soils of Punjab. For a permanent removal of selenite from drinking water biofiltration columns might be applied in small-scale or huge biofilters in large-scale to precipitate selenite from the raw water source as elemental Se. However, further investigations are necessary to find out possible interactions of selenite-reducing bacteria with other soil bacteria for ion removal.

## Methods

### Sampling

Based upon our previous survey [4], Jainpur village (N31°08.082’ E076°11.776) located in north-eastern part of Punjab, India was selected for soil sampling in March 2011, as we found high Se concentrations in those soils. The soil samples were transferred to the laboratory in sealed plastic bags and kept at 4°C until further use. The top layer from 0–2 cm of soil profiles was selected for preparing soil slurries for isolation of bacteria under aerobic conditions.

### Bacterial isolation and identification

Soil slurry was prepared by suspending 5 g soil from the top layer in 100 ml tap water. Twenty ml portions of slurry were incubated in 100 ml Erlenmeyer flasks under gentle shaking overnight at 28 ± 2°C. From this slurry a mixed bacterial culture capable of reducing selenite was enriched by multiple transfers of 2.5% of the initial soil suspension and later on of the cell suspension to enrichment medium (EM), described by Ghosh et al. [29]. To this medium 133.2 mg/l of Na_2_SeO_3_^.^5H_2_O (= 40 mg/l Se (IV)) and 1 g/l glucose monohydrate as the main carbon source were added. The selenium concentration in EM was increased step wise up to concentrations of 160 mg/l of Se (IV) by adding the respective amount of Na_2_SeO_3_^.^5H_2_O (up to 533 mg/l). Before each Se increment, bacterial activity was monitored visually (red color formation of elemental Se in EM) and by measuring Se (IV) reduction with an ion chromatograph (IC). Four strains of bacteria were isolated from the Se (IV) reducing enrichment culture by picking single colonies from Petri dishes that contained 160 mg/l Se (IV) in EM-Medium and 1.5% agar. Selenite reduction was indicated by a red color of the colonies. The colonies were picked and re-grown in liquid EM containing 160 mg/l Se (IV). The reduction of Se (IV) was confirmed again by red coloration of the medium and by measuring selenite reduction with IC. The culture suspension was streaked once more onto agar plates and single colonies were again inoculated into liquid medium. This procedure was repeated a third time to ensure purity of the cultures.

DNA extraction from cells of the four selected strains was performed with chloroform-phenol. Universal eubacterial 16 S rDNA sequencing primers 27 F (5-AGAGTTT GATCCTGGCTCAG-3) and 1492R (5-GGTTACCTTGT TACGACTT-3) [34] were used for identification of the strains. The amplification of the 16 S rDNA gene by polymerase chain reaction (PCR) was carried out in a Biometra Thermocycler T Gradient. The amplified products were sent for DNA-sequencing to Seqlab Laboratories, Göttingen, Germany. The resulting nucleotide sequences were compared with known sequences of the database at National Center for Biotechnology Information (NCBI) by using Basic Local Alignment Search Tool (BLAST). Sequence alignment analyses of the four strains were conducted using MEGA4 [35]. Details of the methods for DNA isolation and molecular identification of isolates have been described elsewhere [36]. For morphological characterization of the four isolates a 1000 x magnification phase contrast microscope (Zeiss Axioskop, Göttingen, Germany) was used. Physiological and biochemical tests were performed as described in Bergey’s Manual [26]. Production of acids from sugars was tested at 27°C after incubation of cultures for 1 day according to Hugh and Leifson [37]. Growth of cultures on liquid and agar media was tested by incubation at 27°C for up to 7 days. Other biochemical assays (Table 1) were performed using microplates and the Micronaut-IDS/STREP 2 identification system (Merlin diagnostics GmbH, Bornheim-Hersel,Germany) following the manufacturer’s instructions. All biochemicals were of microbiological grade and were purchased from Carl Roth (Karlsruhe), Merck (Darmstadt) or Fluka (Steinheim), Germany.

### Selenite reduction assay

All assays to investigate selenite reduction by mixed or pure microbial cultures were carried out in duplicate in 250 ml Erlenmeyer flasks containing 100 ml enrichment medium (EM). The flasks were incubated on a shaker at 110 rpm and 28 ± 2°C. The different concentrations of selenite or selenate in the assays were obtained by adding the required volume of stock solutions of 13.32 g/l Na_2_SeO_3_^.^5H_2_O (= 4 g/l Se IV) or 4.78 g/l Na_2_SeO_4_ (= 2 g/l Se VI). No inoculum was added to sterile controls. For other assays the percentage of the inocula was as indicated in the results.

### Analysis

Selenite concentrations in the samples were determined with an ion chromatograph (Dionex ICS-90) employing an AS9-HC 4 mm × 250 mm (IonPac®) analytical column. The eluent was 9 mM Na_2_CO_3_ and H_2_SO_4_ acid was used as a regenerate. The sample volume was 1.2-1.5 ml. Bacteria and other particles were pelleted by centrifugation at 6700 g for 7–8 min. The supernatant was once more centrifuged at 9660 g for 4–5 minutes in a Microfuge (Eppendorf, Hamburg) before analysis. All chemicals used for analyses were of analytical grade and were purchased from Merck/VWR (Darmstadt) or Carl Roth (Karlsruhe), Germany. The optical density of the cell suspension was measured at 578 nm using an UV LKB Biochrom Ultrospec II spectrophotometer (Cambridge, United Kingdom). The samples for scanning electron microscopy (SEM) + energy-dispersive X-ray spectroscopy (EDX) were prepared by mounting them on silicon wafers (Plano, Wetzler, Germany). For transmission electron microscopy (TEM) + EDX), formvar-coated copper grids 200 mesh (Plano, Wetzler, Germany) were used as a sample support. SEM was performed using a LEO 1530 Gemini microscope with a Schottky field emitter and TEM with a Philips CM 200 FEG/ST microscope. Electron microscopy was done by the Laboratory for Electron Microscopy (LEM) at Karlsruhe Institute of Technology, Germany.

## Competing interests

The authors declare that they have no competing interests.

## Authors’ contributions

MB participated in sample collection, designed and carried out the research work and drafted the manuscript. SS did the molecular biological investigations. JW was involved in the conception and interpretation of the research and contributed to finalize the manuscript. The authors have read and approved the final manuscript.
